# OS-PSO: A Modified Ratio of Exponentially Weighted Averages-Based Optical and SAR Image Registration

**DOI:** 10.3390/s24185959

**Published:** 2024-09-13

**Authors:** Hui Zhang, Yu Song, Jingfang Hu, Yansheng Li, Yang Li, Guowei Gao

**Affiliations:** 1 Beijing Key Laboratory of Sensor, Beijing Information Science & Technology University, Beijing 100101, China; 18438387221@163.com (H.Z.); jf_1213@163.com (J.H.); shanshui4956@126.com (Y.L.); ggw@bistu.edu.cn (G.G.); 2 Key Laboratory of Modern Measurement and Control Technology, Ministry of Education, Beijing Information Science and Technology University, Beijing 100192, China; 3 State Key Laboratory of Transducer Technology, Aerospace Information Research Institute, Chinese Academy of Sciences, Beijing 100190, China; liyang.cas@aircas.ac.cn

**Keywords:** image registration, SIFT algorithm, MROEWA, enhanced matching method

## Abstract

Optical and synthetic aperture radar (SAR) images exhibit non-negligible intensity differences due to their unique imaging mechanisms, which makes it difficult for classical SIFT-based algorithms to obtain sufficiently correct correspondences when processing the registration of these two types of images. To tackle this problem, an accurate optical and SAR image registration algorithm based on the SIFT algorithm (OS-PSO) is proposed. First, a modified ratio of exponentially weighted averages (MROEWA) operator is introduced to resolve the sudden dark patches in SAR images, thus generating more consistent gradients between optical and SAR images. Next, we innovatively construct the Harris scale space to replace the traditional difference in the Gaussian (DoG) scale space, identify repeatable key-points by searching for local maxima, and perform localization refinement on the identified key-points to improve their accuracy. Immediately after that, the gradient location orientation histogram (GLOH) method is adopted to construct the feature descriptors. Finally, we propose an enhanced matching method. The transformed relation is obtained in the initial matching stage using the nearest neighbor distance ratio (NNDR) and fast sample consensus (FSC) methods. And the re-matching takes into account the location, scale, and main direction of key-points to increase the number of correctly corresponding points. The proposed OS-PSO algorithm has been implemented on the Gaofen and Sentinel series with excellent results. The superior performance of the designed registration system can also be applied in complex scenarios, including urban, suburban, river, farmland, and lake areas, with more efficiency and accuracy than the state-of-the-art methods based on the WHU-OPT-SAR dataset and the BISTU-OPT-SAR dataset.

## 1. Introduction

With the continuous advancement of remote sensing technology, both domestically and internationally, the variety of remote sensing images has become increasingly di-verse. The optical image, containing a wealth of spectral information and distinctive structural features, is easy to understand. Meanwhile, the optical image is restricted by adverse weather conditions, such as fog, drizzle, and snow [1]. While synthetic aperture radar (SAR) provides structural details of the reflective target according to the strong penetration ability, the primary influence for the application of SAR images is hindered by speckle noise [2,3]. To overcome the respective limitations of optical and SAR images, their fusion has become an important research direction in the field of remote sensing [4]. The fusion of optical and SAR images aims to combine the advantages of both to achieve all-weather, high-precision remote sensing observations. Image fusion includes image preprocessing, image registration, fusion strategy selection, fusion result optimization, and evaluation. Overall, image registration plays a crucial role for the preprocess of the image fusion [5,6], which ensures that multiple images from different sensors at different times can be precisely aligned in spatial positions before fusion [7,8]. Recently, remote sensing image registration has been extensively researched and broadly categorized into area-based methods and feature-based methods [9].

Area-based methods create a template in the sensed image and then apply multiple similarity measures to find the best correspondences in the reference image. The common similarity measures include mutual information [10] and the normalized cross-correlation [11]. Note that area-based methods are affected by the presence of extremes in the calculation of local features and a large amount of computation.

Compared with area-based methods, feature-based methods perform the registration by extracting image features, thus avoiding a large number of pixel-level computations [12]. The feature-based approach consists of the following four main steps: feature detection, feature description, feature matching, and transform model estimation [13]. The scale invariant feature transform (SIFT) algorithm is a classic feature-based approach that is widely applied for its ability to extract feature descriptors with scale, illumination, and rotation invariance from images [14]. However, when the SIFT algorithm is applied to optical and SAR image registration, the SIFT algorithm still faces two main problems: one is the influence of the high noise levels in SAR images on the stability of the algorithm, and the other is the sensitivity to significant radiometric differences between optical and SAR images. To address these issues, researchers have proposed various extensions of SIFT algorithms, including SAR-SIFT [15], PSO-SIFT [16], and OS-SIFT [17]. Furthermore, some structural feature-based algorithms are gradually applied to optical and SAR image registration due to their low sensitivity to nonlinear radiometric variations [18]. Fan et al. [19] proposed an innovative technique for optical and SAR image registration. The technique is based on the phase congruency structural descriptor (PCSD) and the nonlinear diffusion method, which significantly improves the registration accuracy between SAR and optical images by effectively reducing the interference of speckle noise and enhancing the robustness of the structure descriptor. Zhu et al. [20] proposed a Gabor structure-based registration method. First, the geometric differences between images were eliminated through coarse registration, and then, a more accurate correspondence was obtained via fine registration. Li et al. [21] proposed an image registration algorithm named radiation-variation insensitive feature transform (RIFT) to overcome nonlinear radiation distortions by exploiting the maximum index map and phase consistency. Xiong et al. [22] defined a novel adjacent self-similarity (ASS) feature for efficiently capturing image structures and presented a robust algorithm for registering optical and SAR images by using the ASS feature. Although the above methods can solve the problems of geometric and nonlinear radiometric differences between optical and SAR images, as well as speckle noise in SAR images, to some extent, they still face challenges in the pursuit of high registration accuracy and sufficiently correct correspondences.

Besides classical registration methods, deep learning methods have shown significant advantages in the field of image registration. The advantages are mainly reflected in the extraction of high-level semantic features [23]. These features can accurately capture the structural information of the images and are particularly suitable for SAR and optical image registration. Another major advantage of deep learning methods is their powerful learning and generalization capabilities. Through training on large-scale datasets, the model is able to learn the intrinsic laws and patterns of image matching and apply them to new image data. This makes deep learning methods more flexible and adaptable in dealing with complex and variable image registration tasks. Two-channel networks, Siamese networks, and pseudo-Siamese networks are common deep learning approaches [24]. Wu et al. [25] proposed a deep learning network based on U-net and Siamese networks for SAR and optical image registration. Pseudo-Siamese networks exhibited greater flexibility in feature extraction. Zhou et al. [26] generated multiscale convolutional gradient features (MCGFs) by extracting multidirectional gradient features and constructing a shallow pseudo-Siamese network. Xiang et al. [27] proposed an optical and SAR image registration method combining a pseudo-Siamese network and a residual denoising network, which outperformed the traditional registration algorithm in terms of both computational cost and matching accuracy. Li et al. [28] proposed combining a dual-channel network and a multi-level key-point detector to improve the registration performance by suppressing the geometric and nonlinear radiometric variation between optical and SAR images. In optical and SAR image registration, the deep learning methods still face several challenges despite some progress. First, the obvious geometric and radiometric differences between optical and SAR images disable the traditional Siamese network in multimodal image matching. Although the pseudo-Siamese network is flexible, the distinct two-parameter design increases the risk of overfitting. Second, neural networks are highly dependent on data, and the quality and quantity of training data directly affect network performance. Therefore, the traditional image registration method is still the mainstream direction at this stage.

To improve the number of correct correspondences in image registration, this paper proposes an optical and SAR image registration algorithm based on the SIFT algorithm (OS-PSO). In addition, we construct a novel dataset consisting of Sentinel-1 SAR images and Sentinel-2 optical images in combination with the public WHU-OPT-SAR dataset to evaluate the performance of the proposed algorithm in different satellite images.

The scientific contributions of this paper are as follows:

- We propose a novel optical and SAR image registration approach based on the OS-SIFT. A modified ratio of exponentially weighted averages (MROEWA) operator is designed for the gradient computation of SAR images, aiming to eliminate the problem of the sharp increase in the gradient magnitude at edge points due to sudden dark patches, so that the gradient distributions of SAR images are more consistent with the gradient distributions of optical images computed using the multiscale Sobel operator.
- An enhanced matching method is introduced that defines a novel matching distance based on the scale, position, and main orientation of the key-points for more accurate correspondences.
- We construct an optical and SAR image dataset named BISTU-OPT-SAR and validate the different performances of the OS-PSO algorithm in Sentinel and Gaofen images in combination with the public WHU-OPT-SAR dataset [29]. Furthermore, the performance of the OS-PSO algorithm in different scenarios, such as urban, suburban, river, farmland, and lake, is thoroughly discussed and analyzed.

## 2. Related Works

In this section, we describe an overview of the OS-SIFT algorithm [17], which consist of four critical parts: gradient computation, key-point detection, description extraction, and key-point matching.

### 2.1. Gradient Computation

The optical and SAR images have considerable intensity differences due to their different imaging mechanisms and processing techniques. If the same gradient operator is used for both images, this non-negligible intensity difference might interfere with the accurate calculation of the gradient orientation and magnitude, thereby decreasing the accuracy of the key-point detector in extracting stable features. To ensure the consistency of the edge structure, the OS-SIFT algorithm combines the multiscale Sobel operator and the multiscale ROEWA operator to compute the optical and SAR image gradients [30]. The multiscale Sobel operator, an extension of the traditional Sobel operator, adapts to the needs of image processing at different scales and effectively extracts the structural information of the optical images. Meanwhile, considering that SAR images are sensitive to speckle noise and the traditional gradient operator may produce erroneous gradient estimation in highly reflective areas, the OS-SIFT algorithm employs the multiscale ROEWA operator to calculate the horizontal and vertical gradients, which are defined as follows:

(1) $$R_{h,\delta_{j}}=\frac{M_{u,\delta_{j}}}{M_{d,\delta_{j}}},\;R_{v,\delta_{j}}=\frac{M_{r,\delta_{j}}}{M_{l,\delta_{j}}}$$

(2) $$M_{u,\delta_{j}}=\sum_{x=-M/2}^{M/2}\sum_{y=1}^{N/2}I(p+x,q+y)\exp(-\frac{\left|x|+|y\right|}{\delta_{j}})$$

(3) $$M_{d,\delta_{j}}=\sum_{x=-M/2}^{M/2}\sum_{y=-N/2}^{-1}I(p+x,q+y)\exp(-\frac{\left|x|+|y\right|}{\delta_{j}})$$

(4) $$M_{r,\delta_{j}}=\sum_{x=1}^{M/2}\sum_{y=-N/2}^{N/2}I(p+x,q+y)\exp(-\frac{\left|x|+|y\right|}{\delta_{j}})$$

(5) $$M_{l,\delta_{j}}=\sum_{x=-M/2}^{-1}\sum_{y=-N/2}^{N/2}I(p+x,q+y)\exp(-\frac{\left|x|+|y\right|}{\delta_{j}})$$

where $M_{u,\delta_{j}}$, $M_{d,\delta_{j}}$, $M_{l,\delta_{j}}$ and $M_{r,\delta_{j}}$ represent the local exponentially weighted average of any pixel point from the SAR image in the four directions, up, down, left, and right, respectively. (p,q) is the position of the pixel point. M and N represent the size of the processing window (related to the scale parameter δ), and I is the pixel intensity. The horizontal and vertical gradients at any pixel point in the SAR images are calculated as follows:

(6) $$G_{x,\delta_{j}}=\log(R_{h,\delta_{j}})$$

(7) $$G_{y,\delta_{j}}=\log(R_{v,\delta_{j}})$$

The magnitude and orientation of the gradient at each pixel point can be determined by using the gradient components computed in the horizontal and vertical directions in the SAR image as follows:

(8) $$G_{m}=\sqrt{{\left(G_{x,\delta_{j}}\right)}^{2}+{\left(G_{y,\delta_{j}}\right)}^{2}}$$

(9) $$G_{o}=\arctan(G_{x,\delta_{j}}/G_{y,\delta_{j}})$$

the scale parameter $\delta_{j}$ satisfies the condition $\delta_{j+1}=k\delta_{j}$. In the gradient computation of the SAR image, the weighting effect of the exponential function reduces noise interference and ensures reliable gradient computation.

### 2.2. Key-Point Detection

After calculating the coherent gradients, the OS-SIFT algorithm constructs two Harris scale spaces [31] for accurate the detection of repeatable key-points. In addition, the algorithm is also capable of localization refinement of the detected key-points, which enhances the accuracy of key-point detection in optical and SAR images.

### 2.3. Descriptor Extraction

The OS-SIFT algorithm adopts the GLOH method [32] to construct descriptors and assign dominant orientations. The consistency of the gradient orientation of the optical and the SAR images must be ensured during the process of assigning the dominant orientations. The OS-SIFT algorithm restricts the orientation of these images to the interval [0,180°] to avoid 180° changes in the gradient orientation due to the gradient reversal. In constructing the descriptor, the GLOH method first divides the surroundings of the key-points into three concentric circles of different radii, each of which is further subdivided into 17 sub-regions to completely cover the structure around the key-points. The GLOH method selects eight uniformly distributed orientations within each sub-region for calculating the gradient magnitude to capture the detailed gradient variations. Subsequently, this gradient information is combined into a gradient histogram, where each sub-region corresponds to a histogram and each bin represents a specified gradient orientation. The value of the bin is derived by summing the gradient magnitude of all pixels in that orientation. The peaks of the histogram represent the dominant orientations. The gradient histograms of all the sub-regions are combined to form a high-dimensional feature vector, which is the descriptor of the key-points.

### 2.4. Key-Point Matching

The most commonly used matching strategy for the OS-SIFT algorithm is the nearest neighbor distance ratio (NNDR) method [33], which filters out unstable matches by setting an appropriate threshold for the ratio of the closest distance of a descriptor to the second closest distance. To obtain more correct correspondences, an enhanced RANSAC algorithm, namely the fast sample consensus (FSC) algorithm [33], is proposed to remove outliers. The FSC algorithm effectively eliminates most false correspondences by iteratively estimating the transformation model and checking each key-point to determine whether it matches the model.

## 3. The Proposed Registration Framework

**Figure 1:**
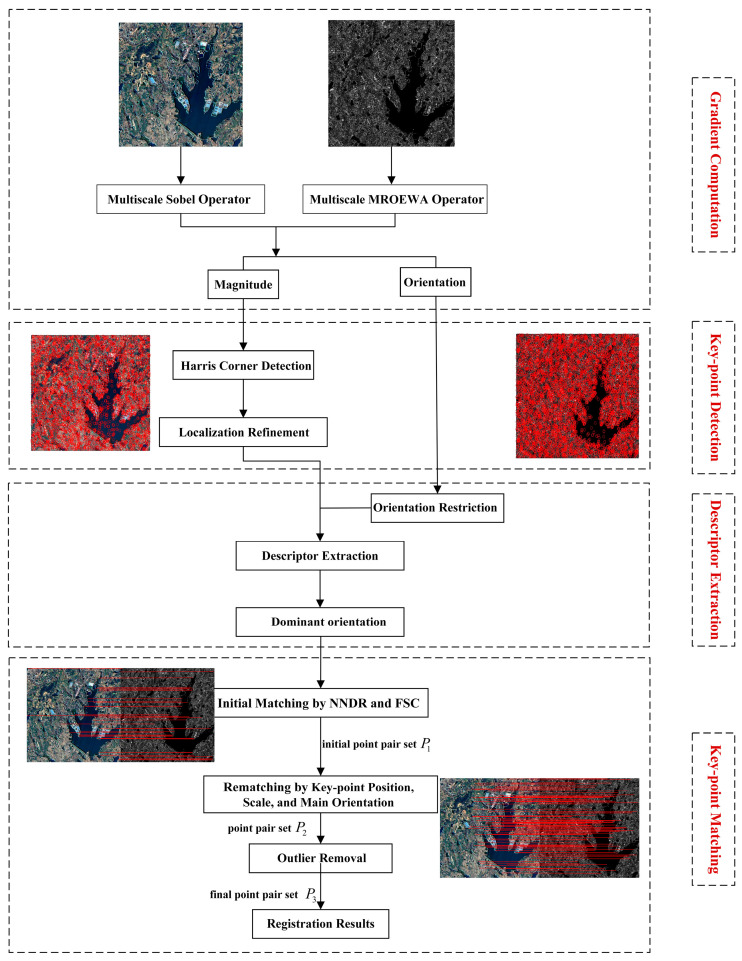
Overall framework of our proposed OS-PSO algorithm.

In this section, we describe the innovative modules introduced based on the OS-SIFT algorithm, including the MROEWA operator for SAR images and the enhanced matching method [16,34]. The flowchart of the proposed OS-PSO algorithm is illustrated in Figure 1.

### 3.1. MROEWA Operator

**Figure 2:**
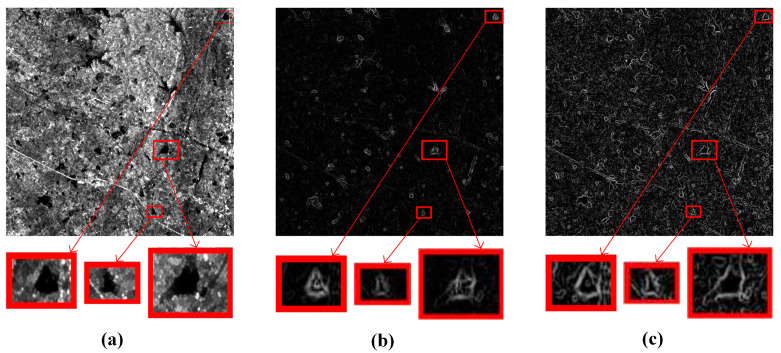
Illustrations of gradient images with dark patches at the same scale. (**a**) SAR image with dark patches; (**b**) ROEWA-processed SAR image; (**c**) MROEWA-processed SAR image.

The multiscale ROEWA operator is used in the gradient computation of SAR images due to its excellent ability to suppress speckle noise. However, the filtering results of the local filtering at the edge points ($M_{d,\delta_{j}}$ and $M_{l,\delta_{j}}$) are likely to approach zero, and the filtering results in the other directions remain normal when the SAR image exists as dark patches. This phenomenon eventually leads to a dramatic increase in the horizontal and vertical gradient of the SAR image, forming a distinct difference with other pixel points. From Figure 2a, we can see the obvious dark patches in the boxes.

To solve the effect of dark patches on the gradient computation of SAR images and narrow the difference in gradient distribution between SAR and optical images, a novel MROEWA operator is used for the linear processing of local filtering results of SAR images. The definition of MROEWA is as follows:

(10) $$R_{h,\delta_{j}}^{*}=\frac{M_{u,\delta_{j}}+q}{M_{d,\delta_{j}}+q}$$

(11) $$R_{v,\delta_{j}}^{*}=\frac{M_{r,\delta_{j}}+q}{M_{l,\delta_{j}}+q}$$

where q represents the linear processing parameter, generally in the range of [0, 1], which can be adjusted to various datasets. To optimize the registration performance of the MROEWA operator on different types of images, the linear processing parameter q is adjusted based on the sensitivity analyses of different datasets.

**Figure 3:**
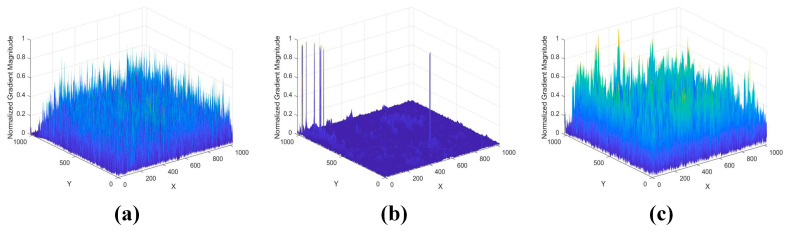
Impact of dark patches on gradient magnitudes at the same scale. (**a**) Sobel operator in the optical image; (**b**) ROEWA operator in the SAR image. (**c**) MROEWA operator in the SAR image.

Figure 3 illustrates the gradient magnitude of the optical and SAR images with sudden dark patches. The optical image mainly relies on visible or infrared light imaging, and the characteristics of these light bands enable the optical image to maintain high clarity and contrast, which can visualize the surface details of objects. Consequently, the optical image is generally not disturbed by dark patches, and the gradient of the optical image does not show any substantial peaks, as shown in Figure 3a. The ROEWA operator reveals pronounced peaks at the location of dark patches within the SAR image. The anomalies underscore the sensitivity of the ROEWA operator to dark patches. The MROEWA operator demonstrated does not show excessive peaks in response to these dark patches compared to the ROEWA operator. This characteristic translates into a reduction in the difference in gradient magnitude between the dark patches and the surroundings, leading to a more robust representation and a more consistent gradient distribution between optical and SAR images. On this basis, we utilize the consistent gradients of optical and SAR images to construct the Harris scale space for key-point detection and further construct descriptors using the GLOH method.

### 3.2. Enhanced Feature Matching

We propose an enhanced matching algorithm that utilizes the inherent information (scale, position, and main orientation) of each key-point to obtain more accurate correspondences. Two key-point sets, $P_{r}=\{p_{r,1},p_{r,2},p_{r,3}\ldots \ldots \}$ and $P_{s}=\{p_{s,1},p_{s,2},p_{s,3}\ldots \ldots \}$, are extracted from the reference and sensed images, respectively, where $\left(x_{r,i}{,\;y}_{r,i}\right)$, $s_{r,i}$, and $\theta_{r,i}$ are the position, scale, and main orientation of the key-point $p_{r,i}$ in the reference images and $\left(x_{s,j},y_{s,j}\right)$, $s_{r,j}$, and $\theta_{s,j}$ are the position, scale, and main orientation of the key-point $p_{s,j}$ in the sensed images. The position transformation error, scale error, and main orientation error of the key-points $p_{r,i}$ and $p_{s,j}$ are defined as follows:

(12) $$e_{P}(p_{r,i},p_{s,j})=||(x_{r,i},y_{r,i})-T((x_{s,j},y_{s,j}),\varphi )||$$

(13) $$e_{s}(p_{r,i},p_{s,j})=abs(1-(r_{\mathrm{mod}e})\frac{s_{s,j}}{s_{r,i}})$$

(14) $$e_{o}(p_{r,i},p_{s,j})=\left|\theta_{r,i}-\theta_{s,j}-\Delta \theta_{\mathrm{mod}e}\right|$$

where $T((x_{s,j},y_{s,j}),\varphi )$ is a similarity transformation model and φ is the transformation model parameter. $r_{\mathrm{mod}e}$ denotes the scale ratio between the reference and sensed images. A composite distance metric, called the position scale orientation Euclidean distance ($ED_{PSO}$), is introduced to evaluate these differences more comprehensively. The $ED_{PSO}$ is computed as follows:

(15) $$ED_{PSO}\left(p_{r,i},p_{s,j}\right)=(1+e_{p}(p_{r,i},p_{s,j}))(1+e_{s}(p_{r,i},p_{s,j}))(1+e_{o}(p_{r,i},p_{s,j}))ED_{0}(p_{r,i},p_{s,j})$$

where $ED_{0}(p_{r,i},p_{s,j})$ represents the Euclidean distance of the descriptors between key- points $p_{r,i}$ and $p_{s,j}$, the $ED_{PSO}$ takes the minimum value when the key-points of the reference and sensed images are correctly matched, and the following are the specific matching steps:

(1) Initial matching: First, the initial point set $P_{1}$ is obtained using the NNDR method, and the ratio threshold is set to $t_{1}$. The next step is to generate histograms of the scale ratio, main orientation difference, horizontal shifts, and vertical shifts. Finally, the FSC algorithm is employed to compute the initial transform parameter φ from the initial point set $P_{1}$, which is a reliable parameter estimation method to accurately estimate the transform parameters under speckle noise.

(2) Re-matching: Based on the initial matching, $ED_{PSO}$ is designed as the distance metric for re-matching to enhance the accuracy and robustness of the matching results. We identify and remove false correspondences by comparing the ratio of composite distances between the nearest and second nearest neighbors of each key-point with a pre-defined ratio threshold $t_{2}$, thus obtaining a new point set $P_{2}$. However, there may be several outliers contained in the point set $P_{2}$. The point set $P_{3}$ is obtained by removing outliers using Equations (16) and (17). $\left(x_{r,1},y_{r,1}\right)$ and $\left(x_{s,1},y_{s,1}\right)$ denote the coordinates of the corresponding key-points in the point set $P_{2}$.

(16) $$|x_{r,1}-r_{\mathrm{mod}e}(x_{s,1}*\cos(\Delta \theta_{\mathrm{mod}e})-y_{s,1}*\sin(\Delta \theta_{\mathrm{mod}e}))-\Delta x_{\mathrm{mod}e}|\geq \Delta x_{t}$$

(17) $$|y_{r,1}-r_{\mathrm{mod}e}(x_{s,1}*\sin(\Delta \theta_{\mathrm{mod}e})+y_{s,1}*\cos(\Delta \theta_{\mathrm{mod}e}))-\Delta y_{\mathrm{mod}e}|\geq \Delta y_{t}$$

where $\Delta x_{t}$ and $\Delta y_{t}$ represent the thresholds for horizontal and vertical differences, respectively. These thresholds are set to the bin widths of the corresponding histograms.

## 4. Experimental Results and Discussion

This section utilizes two datasets, BISTU-OPT-SAR and WHU-OPT-SAR [29], to evaluate the full effectiveness of the proposed OS-PSO algorithm. First, we introduce the characteristics of these two experimental datasets, the parameter settings, and evaluation criteria of the experiments. Then, we evaluate the performance of the OS-PSO algorithm in terms of key-point detection and enhanced matching, aiming to find the parameter configurations with the optimal registration results. Next, the robustness of the OS-PSO algorithm in the face of geometric differences is discussed. Finally, the adaptability of the OS-PSO algorithm and existing algorithms in various complex scenarios is compared and analyzed. All experiments are performed on a computer with an AMD R7-6800H processor and 16.0 GB of RAM using MATLAB R2018a software.

### 4.1. Description of the Datasets and Parameter Settings

To fully evaluate the proposed OS-PSO algorithm, two datasets are selected for testing: the self-made BISTU-OPT-SAR dataset and the public WHU-OPT-SAR dataset [29], The details of the dataset are shown in Table 1.

The BISTU-OPT-SAR dataset covers parts of the Tianjin and Anhui Province in China, and its data include optical images from the Sentinel-2 satellite and SAR images from the Sentinel-1 satellite. Before testing, the optical and SAR images of the BISTU-OPT-SAR dataset undergo the necessary preprocessing to ensure the accuracy and validity of the following registration work. For Sentinel-2 optical images, we utilize the Sen2cor tool to conduct radiometric calibration and atmospheric correction to remove aberrations in the images caused by light and the atmosphere. For Sentinel-1 SAR images, we use a more complex preprocessing procedure, which mainly includes orbit correction [35], thermal noise removal [36], speckle filtering [37], radiometric calibration [38], and geocoding [39], and all the steps are performed in the SNAP software. Orbital correction removes the effect of orbital errors on the SAR image by accurately measuring and correcting the orbital parameters. Thermal noise removal is used to calibrate the backscatter interference caused by thermal noise in the input SAR data. Speckle filtering is specifically used to reduce speckle noise in the image caused by the coherence of the SAR system. Radiometric calibration is the process of converting the digital values of raw remote sensing images into physical quantities, ensuring that data from different sensors have a consistent scale for the comparison and analysis. Geocoding is the process of converting the pixel information in the SAR images to the actual geographic coordinate system to achieve an accurate match between the SAR images and the real geographic space. After preprocessing, both optical and SAR images are in the WGS84 geographic coordinate system. The image size of Tianjin in the BISTU-OPT-SAR dataset is 1024 × 1024 pixels, and the image size of Anhui Province is 850 × 850 pixels. To ensure consistency between optical and SAR images, the resolution of the optical images is also resampled to 10 m to achieve pixel-by-pixel correspondence with the SAR images.

The WHU-OPT-SAR dataset contains optical images taken by the GF-1 satellite and SAR images captured by the GF-3 satellite, both of which are in the WGS84 geographic coordinate system, with a resolution of 5 m after pre-processing. Considering the efficiency of data processing and analysis, the WHU-OPT-SAR dataset is divided into several smaller image patches, each with a size of 800 × 800 pixels.

All parameters for our proposed OS-PSO algorithm are set following the instructions of the OS-SIFT algorithm and PSO-SIFT algorithm. For Harris scale spaces, we set the scale of the first layer to 2 and the constant k between adjacent scales to $2^{\frac{1}{3}}$. The number of scales is set to 8, and the arbitrary parameter d is 0.04. The setting of the parameter q in the MROEWA operator is obtained on the basis of analyses and adjustments after experiments on several images. By setting $t_{1}$ to 0.95, it ensures that a sufficient number of correspondences are preserved to lay the foundation for further fine processing. To obtain more precise correspondences, the ratio threshold of the re-matching ($t_{2}$) is optimized during the enhanced matching process.

### 4.2. Evaluation Criteria

This paper adopts a dual evaluation system combining subjective and objective methods. The subjective method consists of observing matching images, checkerboard mosaic images, and enlarged sub-images to check the positional information of the matching points and the details of image registration. The objective method uses evaluation criteria, such as the correct match number (CMN), correct match rate (CMR), and root mean square error (RMSE). The registration is judged to have failed if the CMN is less than 4.

The CMR is defined as follows:

(18) $$CMR=\frac{N_{cor}}{N_{total}}$$

where $N_{cor}$ represents the number of correctly matched key-points and $N_{total}$ represents the number of key-points screened using the NNDR method.

The RMSE reflects the accuracy of the algorithm’s registration and is defined as follows:

(19) $$RMSE=\sqrt{\frac{1}{n}\sum_{i=1}^{N}{\left(x_{r,i}-x_{s,i}^{\prime }\right)}^{2}+{\left(y_{r,i}-y_{s,i}^{\prime }\right)}^{2}}$$

where N represents the total number of matching key-points, $\left(x_{r,i},y_{s,i}\right)$ and $\left(x_{r,i}^{\prime },y_{s,i}^{\prime }\right)$ mean the i-th matching key-point pairs. $\left(x_{r,i},y_{s,i}\right)$ is the actual output of the reference images, and $\left(x_{r,i}^{\prime },y_{s,i}^{\prime }\right)$ is the predicted output of the sensed images, respectively.

### 4.3. Experiments on Key-Point Detection

In this section, we use the repeatability rate [40,41] as an indicator to evaluate the performance of the proposed detector. Specifically, any two key-points $\left(x_{r},y_{r}\right)$ and $\left(x_{s},y_{s}\right)$ of a given pair for the reference and sensed image are judged as repeatable key-points if they satisfy the following:

(20) $$\sqrt{{\left(x_{s}-x_{r}\right)}^{2}+{\left(y_{s}-y_{r}\right)}^{2}}<t$$

The localization error (t) is set from 1 to 5 in the proposed OS-PSO algorithm. The repeatability rate is determined by the ratio between the number of repeatable key-points and the total number of key-points. As the repeatability rate increases, key-point detection becomes stable.

The proposed Harris detector based on the MROEWA operator suppresses the potential influence of sudden dark patches based on key-point detection. The in-depth experimental analysis of the linear processing parameters q in the MROEWA operator is carried out. Our goal is to find a linear processing parameter q that makes our proposed detector more stable through continuous adjustments and optimization.

**Figure 4:**
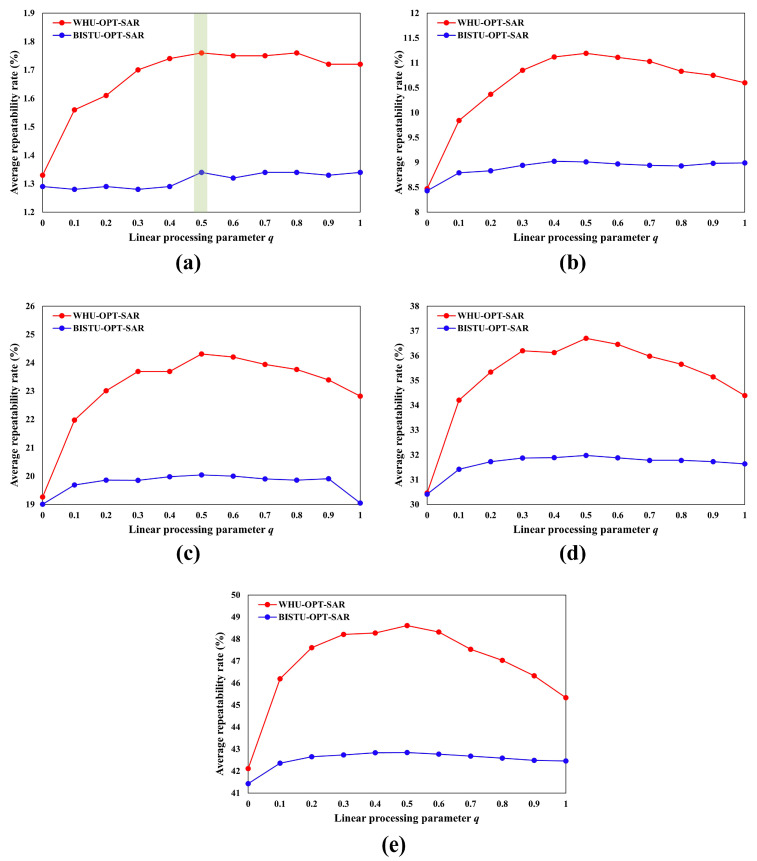
Repeatability of key-points in the BISTU-OPT-SAR dataset and WHU-OPT-SAR dataset with different location errors. (**a**–**e**) represents location error t = {1, 2, 3, 4, 5}.

We calculate the average repeatability rate of ten image pairs for the WHU-OPT-SAR dataset and the BISTU-OPT-SAR dataset with different localization errors (t), respectively, and the experimental results are displayed in Figure 4. It is observed that the proposed method is more outstanding in detection performance compared with the SAR-Harris (q set to 0) detector, and the adjustment of the q has a stronger effect on the WHU-OPT-SAR dataset compared to the BISTU-OPT-SAR dataset. When q = 0.5, our proposed method reaches the highest level of key-point repeatability based on both datasets. The average repeatability rate of the WHU-OPT-SAR dataset is close to 50% when the localization error t is set to 5.

### 4.4. Experiments on Enhanced Matching

**Figure 5:**
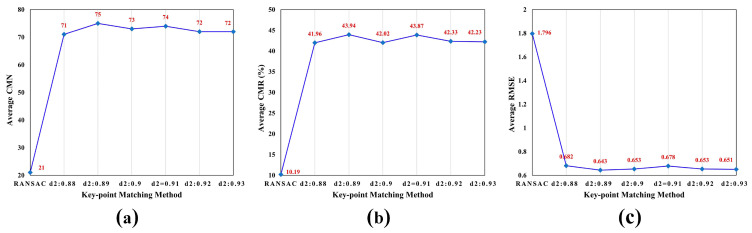
The performance of different key-point matching methods. (**a**) Average CMN; (**b**) average CMR, (**c**) average RMSE.

Our experiments in this part aim to verify the performance of the enhanced matching method and to explore the impact of the ratio threshold ($t_{2}$) for re-matching on the registration results. The first step is to compare the enhanced matching method with the classical RANSAC method, focusing on evaluating its advantages in improving the number of correct correspondences. We select ten image pairs and calculate the metrics, such as the average CMN, average CMR, and average RMSE for different matching methods. Figure 5 illustrates the performance of different feature matching methods. It displays that the enhanced matching algorithm outperforms the RANSAC method in CMN, RMSE, and CMR. Further analysis reveals the superior performance of the enhanced matching method when $t_{2}$ is set to 0.89.

### 4.5. Discussion on the Robustness of Geometric Differences

**Figure 6:**
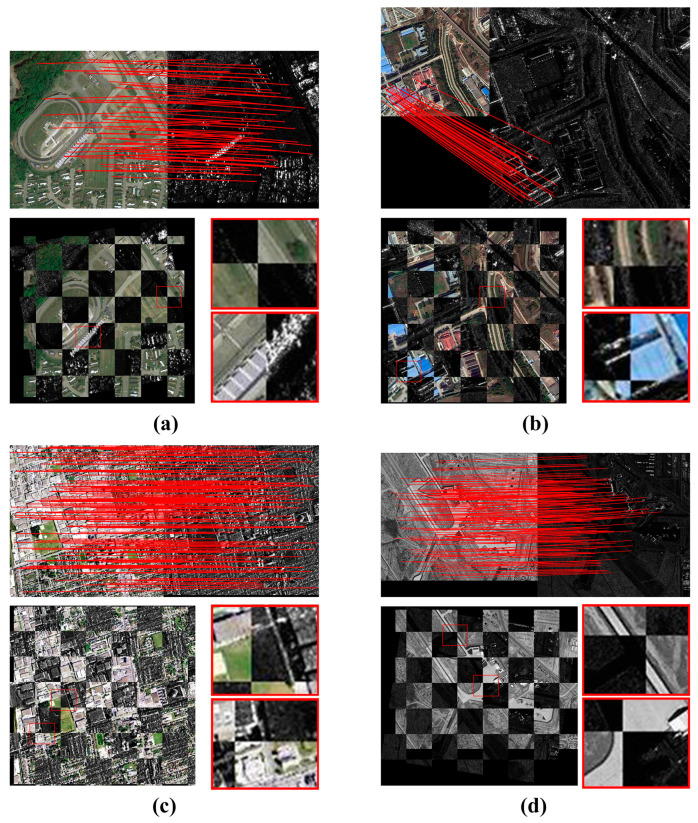
Registration results of the OS-PSO algorithm based on four image pairs with geometric differences. (**a**) P1 with significant rotation differences and slight scale differences; (**b**) P2 with significant scale differences; (**c**) P3 with slight scale and rotation differences; (**d**) P4 with significant translation and rotation differences.

In this section, considering the WHU-OPT-SAR dataset and the BISTU-OPT-SAR dataset with no geometric differences between the optical images and the SAR images, we additionally select four image pairs with different geometric differences for the experiment. Among them, P1 exhibits slight scale differences and obvious rotation differences, P2 has obvious scale differences but no rotation differences, P3 has no obvious differences in both scale and rotation, while P4 has obvious translation and rotation differences. Specific information is provided in Table 2. For different image pairs, the parameters for re-matching need to be fine-tuned to obtain similar RMSE values. The experimental results are shown in Figure 6.

The experimental results demonstrate that the proposed OS-PSO algorithm successfully matches four pairs of optical and SAR images, demonstrating its strong adaptability to diverse geometric differences. Specifically, most of the correctly corresponding points for P1 are located in non-vegetated areas. P2 exhibits significant scale differences, resulting in the lowest number of correct correspondences among the four image pairs, which are primarily concentrated in areas with dense buildings. From the enlarged sub-images of Figure 6b, it can be observed that the errors are significantly smaller around the buildings compared to the road areas. This phenomenon is attributed to the shading effect in the building areas, which reduces the gray scale difference between the optical and SAR images in these areas. P3 has a sufficient number of correct correspondences and is evenly distributed because of its unclear geometric differences and regular structure. Despite the obvious geometric differences in P4, the OS-PSO algorithm is still able to achieve excellent registration results because of the minimal gray-scale differences. Especially in areas with significant features, such as airports, the algorithm demonstrates high accuracy. The precise alignment effect can be observed in the enlarged sub-images of Figure 6d. The overall outcomes show that the OS-PSO algorithm exhibits superior robustness in handling image registration with different types of geometric differences.

### 4.6. Comparative Experiments in Different Scenarios

**Figure 7:**
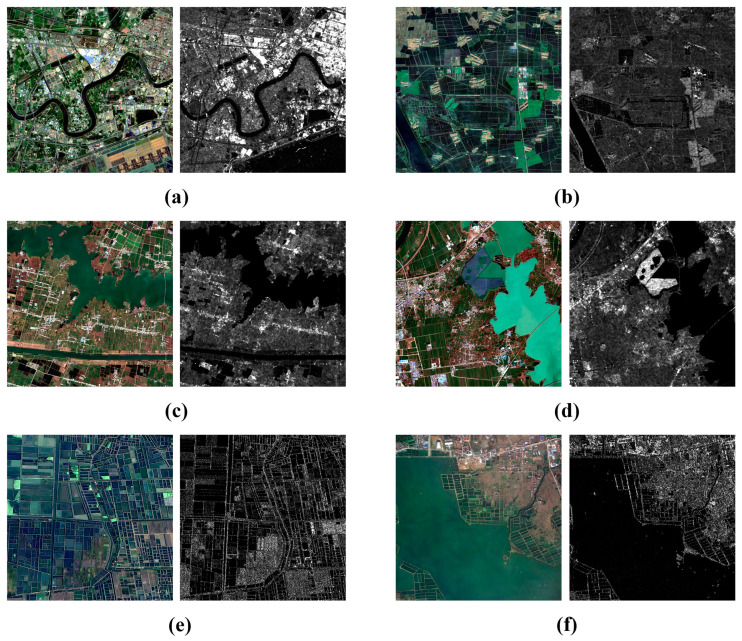
Six image pairs from the BISTU-OPT-SAR and WHU-OPT-SAR datasets. (**a**–**d**) Pairs 1,2,3, and 4 from the BISTU-OPT-SAR dataset; (**e**,**f**) Pairs 5 and 6 are from the WHU-OPT-SAR dataset.

In this section, we select six image pairs from the BISTU-OPT-SAR dataset and the WHU-OPT-SAR dataset to evaluate the performance of our proposed OS-PSO algorithm. These image pairs cover different scenarios, including urban, suburban, river, farmland and lake, as shown in Figure 7. Pairs 1, 2, 3, and 4 are all taken from the BISTU-OPT-SAR dataset. Pairs 1 and 2 show the urban and suburban areas of Tianjin, respectively. Pair 1 contains buildings of different structures with rich structural features, which are more seriously affected by noise and sudden dark patches. Pair 2 consists mainly of vegetation interspersed with a river, which is slightly disturbed by noise. Pairs 3 and 4 are from Anhui Province and show mainly river images; both of them are affected by noise and sudden dark patches to varying degrees. Pair 4 has a significantly gray-scale difference between the optical and SAR images, with the color of the water being lighter in the optical image. Pairs 5 and 6 describe the farmland area and lake area in Hubei Province from the WHU-OPT-SAR dataset. Pair 5 depicts a crisscrossed terraced landscape. Here, the area is flat and has a regular land-use pattern. Pair 6 presents a large lake and scattered villages around it, with a relatively homogenous structure.

Since the proposed OS-PSO algorithm is a feature-based technique, we chose five sophisticated feature-based algorithms for the comparison. SAR-SIFT is an enhanced SIFT algorithm specifically tailored for SAR images. By introducing a new gradient definition, it generates robust directions and magnitudes that are resilient to speckle noise, making it more suitable for feature extraction in SAR images. PSO-SIFT optimizes the computation of image gradients to enhance robustness to intensity differences and introduces enhanced matching methods to increase the number of correct correspondences. OS-SIFT combines the multiscale Sobel operator and the multiscale ROEWA operator to compute the optical and SAR image gradients. RIFT is a novel optical and SAR image registration algorithm that employs phase consistency for key-point detection and maximum index maps for descriptor construction, with rotation and radiation invariance. OSS is an image registration algorithm based on self-similarity features that is robust to radiometric differences between optical and SAR images. In addition, we also design two algorithms, MROEWA+FSC and OS-SIFT+PSO, to further analyze the specific impact of our proposed MROEWA operator and enhanced matching method on the experiments.

Table 3 presents the registration results of the proposed OS-PSO algorithm and the seven comparison methods in different scenarios. These results demonstrate that the OS-PSO algorithm not only successfully achieves image registration for all types of complex scenarios but also outperforms the other algorithms in terms of the number of correct correspondences and the registration accuracy. The RMSE values of the OS-PSO algorithm based on the six pairs of test images are maintained at about 0.7. This excellent performance is attributed to the OS-PSO algorithm’s use of the MROEWA operator and the enhanced matching method. The MROEWA operator is able to suppress the effect of sudden dark patches in the SAR image, thus generating more consistent gradients between the optical and SAR images. The enhanced matching method obtains more correct correspondences by two matching, which can be seen from the comparison of the MROEWA+FSC with the OS-PSO algorithm. In contrast, the PSO-SIFT, SAR-SIFT, and OS-SIFT algorithms fail to register in certain scenarios due to their sensitivity to speckle noise and radiometric differences. The OSS and RIFT algorithms, although successfully achieving registration, are obviously inferior to our proposed OS-PSO algorithm in terms of registration performance, as evidenced by the lower CMN values and higher RMSE values. In addition, these two methods also suffer from the uneven distribution of correct correspondence points, increasing the risk of local mismatch, which is also present in the PSO-SIFT, SAR-SIFT, and OS-SIFT algorithms.

**Figure 8:**
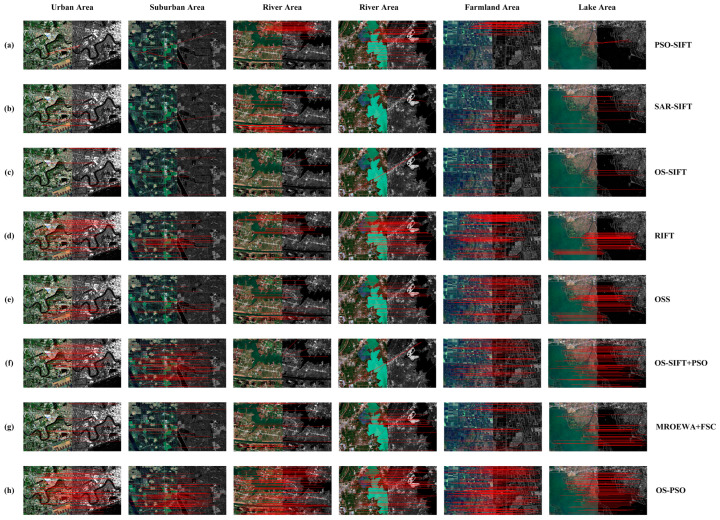
Registration results of different algorithms based on six image pairs from different scenarios, including urban, suburban, river, farmland, and lake. (**a**) PSO-SIFT; (**b**) SAR-SIFT; (**c**) OS-SIFT; (**d**) RIFT; (**e**) OSS; (**f**) MROEWA+FSC; (**g**) OS-SIFT+PSO; (**h**) OS-PSO.

**Figure 9:**
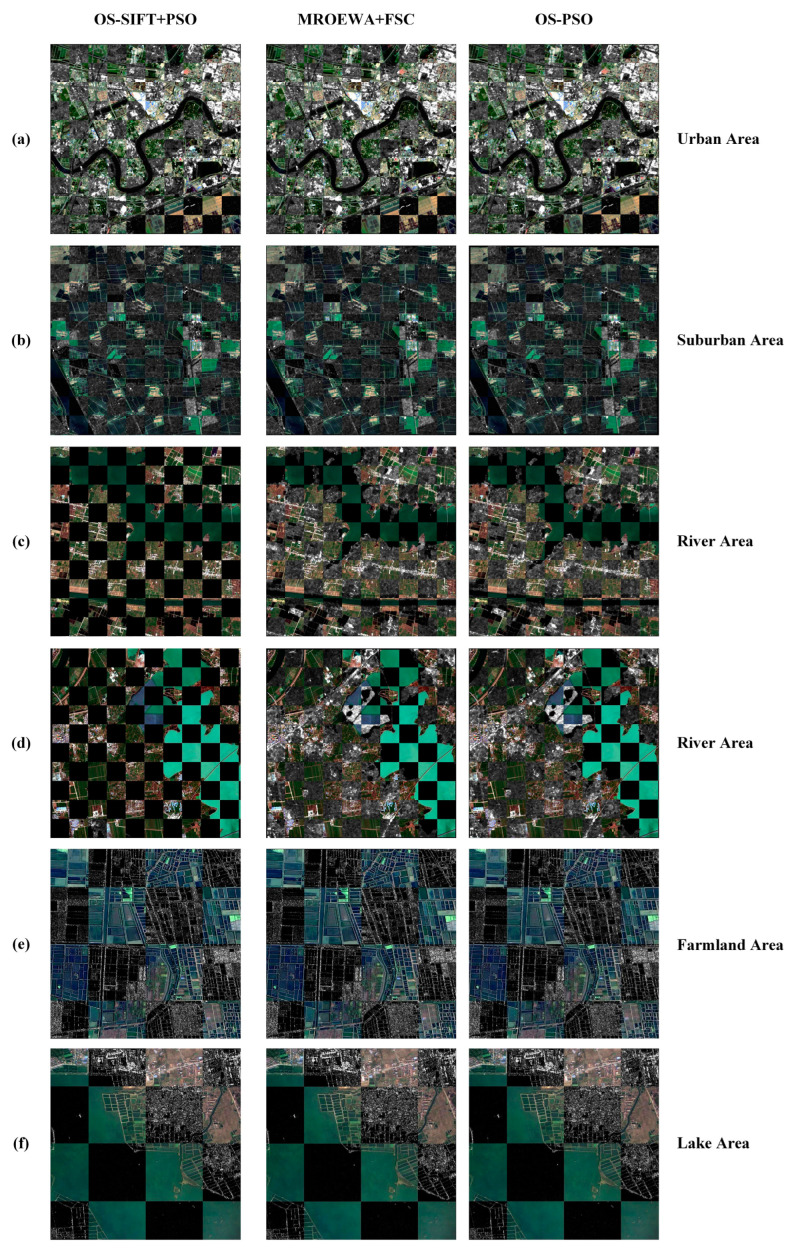
Registration results of OS-SIFT+PSO, MROEWA+FSC, and the proposed OS-PSO algorithms based on six image pairs with different geographic environments. (**a**) Urban Area; (**b**) suburban area; (**c**) river area; (**d**) river area; (**e**) farmland area; (**f**) lake area.

Figure 8 and Figure 9 display the matching images and checkerboard mosaic images of the proposed OS-PSO algorithm based on six pairs of test images. For Pair 1, the PSO-SIFT algorithm fails to achieve the registration because of the interference of severe noise and sudden dark patches, while the other algorithms succeed in the registration task with excellent noise immunity. It is notable that most of the correctly corresponding points are concentrated in areas with dense buildings and around rivers. Figure 9a also demonstrates that these areas are more accurately aligned. Of these, the OS-PSO algorithm demonstrates the most optimal performance in terms of RMSE and CMN, which is attributed to the high repeatability of the key-points. For Pair 2, neither the PSO-SIFT nor SAR-SIFT algorithms are able to detect the correct correspondences due to the unclear structure of the large-area vegetation in the SAR image, thus failing to register the two images. Although the other algorithms succeed in the registration, there are only a few correctly corresponding points in the vegetated areas. A slight deviation of the vegetation area can be seen in Figure 9b. Meanwhile, the CMN values of all methods are lower than Pair 1 except OS-SIFT+PSO.

Pairs 3 and 4 are affected by noise and sudden dark patches to some extent, and the edge structures of the land areas are blurred. In this case, the OS-SIFT algorithm fails to achieve the registration of optical and SAR images because of false correspondences. In Pair 4, there are also obvious gray-scale differences between optical and SAR images, especially in the water area, which pose a great challenge to image registration. Nevertheless, the OS-PSO algorithm successfully registers the optical and SAR images due to the stability of the key-point detector; however, the RMSE value is the largest and the registration accuracy is the lowest among all the image pairs. It is worth noting that most of the correctly corresponding points are located at the land–water boundary, and Figure 9d also demonstrates that more accurate alignments are obtained at the water–land boundary. This is because the land–water boundary, as the intersection of two different textures or gray-scale areas, contains rich edge information that facilitates the detection of key-points.

For Pair 5, the farmland with a regular pattern shows obvious edge and texture features in the remote sensing images, and there is no explicit gray-scale difference between the optical the SAR images, which provide favorable conditions for accurate identification and matching of the corresponding key-points. Therefore, all algorithms successfully achieve the registration. The OS-PSO algorithm identifies and matches 140 correct correspondences, significantly outperforming all the other scenarios. Moreover, the correctly corresponding points of Pair 5 have the most uniform distribution among all image pairs, as shown in Figure 8h, and there is basically no local mismatch. For Pair 6, the lake area presents smooth, single color and texture features, reducing the identifiability of the key-points. It is difficult to capture sufficient and stable key-points during the registration process. As a result, the OS-PSO algorithm has the lowest CMN value of 73 compared to the other five image pairs.

## 5. Conclusions

Aiming at the problem of insufficient correct correspondences caused by different imaging principles between optical and SAR images, this paper proposes a remote sensing image registration algorithm named OS-PSO on the basis of OS-SIFT. First, we design a modified gradient operator based on the ROEWA operator, which provides the uniform linear processing of the local filtering results of SAR images, effectively avoiding the abnormal increase in the gradient magnitude at the edge points of SAR images due to sudden dark patches and thus maintaining the consistent gradient with optical images. Secondly, we establish the Harris scale spaces for optical and SAR images, respectively, and obtain the stable key-points by detecting the local maxima, followed by the descriptor construction utilizing the GLOH method. Then, we introduce an enhanced matching method incorporating the scale, position, and main orientation information of the key-points for re-matching, to increase the number of correspondences. The proposed OS-PSO algorithm significantly improves the number of correct correspondences and the registration accuracy compared with the state-of-the-art algorithms and is applicable to all complex scenarios.

## Figures and Tables

**Table 1:** Detailed description of dataset.

Datasets	Sensor	Resolution (m)	Size (Pixel)	Geographic Location
BISTU-OPT-SAR	Sentinel-2	10	1024 × 1024	Tianjin, China
	Sentinel-1	10	1024 × 1024	
	Sentinel-2	10	850 × 850	Anhui, China
	Sentinel-1	10	850 × 850	
WHU-OPT-SAR	GF-1	5	800 × 800	Hubei, China
	GF-3	5	800 × 800	

**Table 2:** Description of the dataset covering multiple geometric differences.

Pairs	Image Source	Size (pixel)	GSD (m)	Date	Description	Reference
P1	Google Map	520 × 520	0.95	N/A	Image pairs with significant rotation differences and slight scale differences	[22]
	GF-3	500 × 500	1	02/2018		
P2	Google Earth	406 × 406	1.80	11/2016	Image pairs with significant scale differences	[22]
	GF-3	750 × 750	1	11/2016		
P3	Google Earth	528 × 524	3	11/2007	Image pairs with slight scale and rotation differences	[42]
	TerraSAR-X	534 × 524	3	12/2007		
P4	Google Earth	774 × 692	1	10/2012	Image pairs with significant translation and rotation differences	[17]
	TerraSAR-X	900 × 795	1	12/2010		

**Table 3:** Evaluation results (CMN, RMSE) of the proposed OS-PSO algorithm compared with other registration algorithms.

Methods	Evaluation Index	1	2	3	4	5	6
PSO-SIFT	CMN	-	-	43	40	34	-
	RMSE	-	-	1.261	1.400	1.152	-
SAR-SIFT	CMN	12	-	50	-	38	11
	RMSE	1.019	-	1.351	-	1.376	1.137
OS-SIFT	CMN	11	5	-	-	14	7
	RMSE	1.039	1.288	-	-	1.313	1.048
RIFT	CMN	59	37	30	83	106	56
	RMSE	0.849	0.829	0.712	0.920	0.908	0.857
OSS	CMN	32	15	25	28	90	63
	RMSE	1.231	1.211	1.334	1.198	1.149	1.106
OS-SIFT+PSO	CMN	50	80	-	-	65	64
	RMSE	0.662	0.735	-	-	0.672	0.634
MROEWA+FSC	CMN	15	12	22	33	32	37
	RMSE	1.084	1.145	1.096	1.330	1.198	1.345
OS-PSO	CMN	110	83	140	86	170	73
	RMSE	0.693	0.689	0.712	0.705	0.670	0.632

## Data Availability

The BISTU-OPT-SAR dataset and the WHU-OPT-SAR dataset used in this study are publicly available and can be used for research purposes. BISTU-OPT-SAR can be accessed and downloaded from the Github website [https://github.com/zhanghui-1998/BISTU-OPT-SAR, last access: 15 May 2024] and WHU-OPT-SAR can be accessed and downloaded from the Github website [https://github.com/AmberHen/WHU-OPT-SAR-dataset, last access: 15 May 2024.
